# 24-Epibrassinolide (EBR) Confers Tolerance against NaCl Stress in Soybean Plants by Up-Regulating Antioxidant System, Ascorbate-Glutathione Cycle, and Glyoxalase System

**DOI:** 10.3390/biom9110640

**Published:** 2019-10-23

**Authors:** Pravej Alam, Thamer H. Albalawi, Fahad H. Altalayan, Md Afroz Bakht, Mohammad Abass Ahanger, Vaseem Raja, Muhammad Ashraf, Parvaiz Ahmad

**Affiliations:** 1 Department of Biology, College of Science and Humanities, Prince Sattam bin Abdulaziz University, Alkharj 11942, Saudi Arabia; 2 Department of Chemistry, College of Science and Humanities, Prince Sattam bin Abdulaziz University, Alkharj 11942, Saudi Arabia; bakhtpharm@gmail.com; 3 College of Life Science, NorthWest University, Xi’an 710127, China; 4 Department of Botany, Govt. College for Women, Baramulla-193101, Jammu and Kashmir, India; wrajamp2009@gmail.com; 5 University of Agriculture Faisalabad, Faisalabad-38040, Pakistan; ashrafbot@yahoo.com; 6 Botany and Microbiology Department, College of Science, King Saud University, Riyadh 11451, Saudi Arabia; 7 Department of Botany, S.P. College, Srinagar 190001, Jammu and Kashmir, India

**Keywords:** soybean, NaCl stress, 24-epibrassinolide, growth, antioxidants, Asc-Glu cycle, glyoxalase cycle, mineral uptake

## Abstract

The present research was performed to assess the effect of 24-epibrassinolide (EBR) on salt-stressed soybean plants. Salt stress suppressed growth, biomass yield, gas exchange parameters, pigment content, and chlorophyll fluorescence, but all these parameters were up-regulated by EBR supply. Moreover, salt stress increased hydrogen peroxide, malondialdehyde, and electrolyte leakage. EBR supplementation reduced the accumulation of oxidative stress biomarkers. The activities of superoxide dismutase and catalase, and the accumulation of proline, glycinebetaine, total phenols, and total flavonoids increased with NaCl stress, but these attributes further increased with EBR supplementation. The activities of enzymes and the levels of non-enzymatic antioxidants involved in the Asc-Glu cycle also increased with NaCl stress, and further enhancement in these attributes was recorded by EBR supplementation. Salinity elevated the methylglyoxal content, but it was decreased by the EBR supplementation accompanying with up-regulation of the glyoxalase cycle (GlyI and GlyII). Salinity enhanced the Na^+^ uptake in root and shoot coupled with a decrease in uptake of Ca^2+^, K^+^, and P. However, EBR supplementation declined Na^+^ accumulation and promoted the uptake of the aforementioned nutrients. Overall, EBR supplementation regulated the salt tolerance mechanism in soybean plants by modulating osmolytes, activities of key enzymes, and the levels of non-enzymatic antioxidants.

## 1. Introduction

Soil and water pollution due to salinity is responsible for reduced plant growth and crop production around the globe [1,2]. It has been estimated that if salinity persists, it will damage 50% of land before the 21st century [3]. Salinity results in excess accumulation of Na^+^ ion which results in hyperionic and hyperosmotic stresses in plants [4]. These ionic and osmotic stresses impair normal physiological and biochemical processes of the cell thereby contributing considerably to reduced crop production [5]. Increased Na^+^ accumulation in soils affects the soil porosity and reduces soil aeration and water conductance [6]. It also reduces the uptake of water and key mineral elements [6], photosynthesis and pigment synthesis [7], enzyme activity, and secondary metabolite accumulation [4]. Among the key damaging consequences of increased soil salinity is the oxidative damage resulting from excess generation of reactive oxygen species (ROS). Key toxic ROS include superoxide, hydrogen peroxide, and hydroxyl radical, and their excess accumulation has been demonstrated to hamper normal cellular functioning by inducing protein and lipid oxidation resulting in malfunctioning of key cellular organelles including chloroplasts [7,8]. Higher and prolonged salinity stress causes secondary stress, particularly the oxidative stress through triggering enhanced accumulation of ROS. The ROS are highly reactive and can damage the biomolecules and alter their normal functioning [7,8,9].

To protect the plant cells from such harsh environmental stresses, they are equipped with different tolerance mechanisms like (i) compatible osmolyte accumulation, which helps in water uptake and the functioning of other physiological pathways, (ii) partitioning and compartmentalization of toxic ions in vacuoles, and (iii) modulation of antioxidant defense system for the quick removal of ROS [8]. Among the osmolytes, proline, glycine betaine (GB), soluble sugars, amino acids, etc., help in reducing the osmotic stress [8,10]. Ashraf and Foolad [11] reported that the accumulated osmolytes stabilize the membrane integrity and also help in detoxification of toxic ions under NaCl stress. Accumulated osmolytes also prevent polypeptides’ dissociation and protect the photosynthetic apparatus [12] and ribulose-1, 5-bisphosphate carboxylase (Rubisco) activity under NaCl stress [13]. Greater accumulation of compatible osmolytes ameliorates the damaging effects of salinity by improving water uptake as well as it contributes to quick recovery from stress by serving as energy reservoirs [14,15].

In order to lessen the damaging effects of ROS, plants up-regulate the activities of enzymatic antioxidants like, SOD (superoxide dismutase), CAT (catalase), POD (peroxidase), and enzymes of the ascorbate-glutathione (AsA-GSH) cycle [16]. Superoxide dismutase catalyses the conversion of superoxide radicals (O_2_^−^) into molecular oxygen (O_2_) and hydrogen peroxide (H_2_O_2_). The resulting H_2_O_2_ is also deleterious for the cell and is converted to water (H_2_O) and molecular oxygen (O_2_) by the action of CAT [17,18,19]. The enzymatic antioxidant system is supported by non-enzymatic components including ascorbic acid, glutathione, polyphenols, and osmolytes [5,20,21]. To increase the crop production on salinity affected soils, scientists are working day and night to come up with some long-lasting solutions to this problem. One of the sustainable approaches is the external use of plant hormones that could enhance crop production without any harm to the plants.

Among the plant steroids, brassinosteroids (BRs) are known for their role in growth, development, and increased resistance against abiotic stresses [22,23,24]. 24-epibrassinolide (EBR) is one of the forms of brassinolide, being used in different plant studies [25]. Plants receiving EBR externally showed enhanced growth, pigment contents, photosynthetic efficiency, and activities of antioxidants [22,25,26,27,28,29]. Brassinosteroids interact with other key cellular molecules to integrate the tolerance networks for better stress mitigation [30]. For example, external application of EBR enhanced the phenolic compounds and pigments in plants [22,28,31]. In another study, exogenous application of EBR has been demonstrated to avoid the damaging effects of stresses by up-regulating the antioxidant system and osmolyte metabolism [7].

Soybean (*Glycine max*) is cultivated for its high protein source. It is also used as a source of oil and livestock feed. The soybean plant is susceptible to salt toxicity and thus is the reason of its low production under salt stress (REFS). Keeping all this in mind, this research was performed to examine the impact of NaCl and EBR on (a) growth and physio-biochemical attributes, (b) activities/levels of antioxidants and uptake of mineral elements, (c) regulation of AsA-GSH cycle, and (d) glyoxalase cycle.

## 2. Material and Methods

Soybean (*Glycine max*) seeds were immersed for 2 min in 5% NaOCl (sodium hypochlorite) solution and then washed with distilled water. Thereafter, the seeds were allowed to germinate on filter papers in a growth chamber. Healthy seedlings (two per pot) after germination were planted in pots filled with sand and vermicompost (3:1) and were irrigated with Hoagland’s nutrient solution. After one week of seedling growth, salinity stress was imposed by adding NaCl (100 mM) to Hoagland’s solution up to 3 weeks of growth. The unstressed (control) seedlings received Hoagland’s solution only. The nutrient solution consisted of 270 mg N L^−^^1^, 31 mg P L^−^^1^, 234 mg K L^−^^1^, 200 mg Ca L^−^^1^, 64 mg S L^−^^1^, 48 mg Mg L^−^^1^, 2.8 mg Fe L^−^^1^, 0.5 mg Mn L^−^^1^, 0.5 mg B L^−^^1^, 0.02 mg Cu L^−^^1^, 0.05 mg Zn L^−^^1^, 0.01 mg Mo L^−^^1^, and 0.1 mg Na_2_–Fe–ethylenediaminetetraacetic acid (EDTA) L^−^^1^. After 2 weeks of NaCl stress, plants were foliarly sprayed with 10^−7^ mM 24-EBR (20 mL per pot) using Teepol (0.1%) as a surfactant. Stock solution (10^−3^ M) of EBR was prepared and from this stock solution varying concentrations of EBR (10^−5^ M; 10^−7^ M; 10^−9^ M; and 10^−11^ M) were prepared. Supplementation of these concentrations to soybean plants showed varying results and the best results were observed with the concentration of 10^−7^ M. Due to this, we selected this concentration only. The pots were kept in a growth chamber with 26 ± 2 °C/16 ± 2 °C day/night temperature, 70–75% relative humidity, and 18/6 h light/dark photoperiod. Each treatment in the experiment contained five replicates. After 35 days of growth, the plants were uprooted for estimation of different parameters.

### 2.1. Growth Estimation

After harvesting, shoot and root lengths were measured by a scale. Shoot fresh weight (FW) was also recorded by weighing the samples directly. The same samples were kept in an oven for 72 h at 70 °C and then dry weight (DW) measured.

### 2.2. Assessment of Pigment Content and Chlorophyll Fluorescence

Chlorophyll and carotenoid contents in the leaves were determined as per Lichtenthaler and Wellburn [32], the absorbance of the extract was spectrophotometrically read against 80% acetone at 645, 663, 510, and 480 nm for chlorophyll and carotenoids, respectively.

The PAM chlorophyll fluorometer (H. Walz, Effeltrich, Germany) was used for the assessment of chlorophyll fluorescence attributes [33].

### 2.3. Gas Exchange Parameters Estimation

The parameters related to gas exchange such as CO_2_ assimilation rate (*A*), stomatal conductance (*gs*), and transpiration rate (*E*) were analyzed by IRGA (Infrared gas analyzer; LCA-4 model, Analytical Development Company, Hoddesdon, England).

### 2.4. Estimation of Proline and Glycine Betaine (GB)

The assessment of proline content was performed by a procedure given by Bates et al. (1973). The absorbance was measured at 520 nm and proline content presented as μmol g^−1^ fresh weight. The technique of Grieve and Grattan [34] was employed to evaluate the glycine betaine content and the OD was recorded at 365 nm.

### 2.5. Evaluation of Hydrogen Peroxide (H_2_O_2_), Malondialdehyde (MDA), and Electrolyte Leakage (EL)

The procedure of Velikova et al. [35] was used for the determination of H_2_O_2_ and the absorbance was recorded at 390 nm. For the estimation of MDA and EL, the procedures outlined by Madhava Rao and Sresty [36] and Dionisio-Sese and Tobita [37] were followed and EL was calculated by the following formula:

Electrolyte leakage (%) = (EC1 − EC0)/(EC2 − EC0) × 100

### 2.6. Estimation of Antioxidant Enzymes’ Activities and Asc-Glu Cycle

Fresh leaf samples, each 500 mg, were extracted in ice-cold potassium phosphate buffer (100 mM, pH 7.0) and PVP (1%). The mixture was centrifuged at 4 °C for 15 min at 12,000× *g* and the collected material was used for the assay of SOD, CAT, APX, GR, DHAR, and MDHAR.

For the assay of activity of superoxide dismutase (SOD, EC1.15.1.1), the reaction mixture comprising enzyme extract (100 μL), phosphate buffer (100 mM, pH 7.4), riboflavin (50 µM), EDTA (1.0 mM), methionine (10 mM), and NBT (75 µM) was kept for 15 min under fluorescent light. The optical density (OD) was noted at 560 nm and the SOD activity presented as EU mg^−1^ protein.

The activity of catalase (CAT; EC1.11.1.6) was estimated by the method of Aebi [38]. The degradation of H_2_O_2_ was estimated at 240 nm and CAT activity presented as EU mg^−1^ protein.

Ascorbate peroxidase (APX; EC 1.11.1.11) activity as EU mg^−1^ of protein was assayed using the protocol by Nakano and Asada [39] following a reduction in absorbance of the mixture containing hydrogen peroxide and ascorbic acid at 290 nm for 3 min.

The glutathione reductase (GR; EC 1.6.4.2) activity was evaluated by assessing the decreased absorbance of the reaction mixture containing GSSG and NADPH at 340 nm for 3 min, using the method described by Foyer and Halliwell [40], and the activity was measured as EU mg^−1^ of protein.

Monodehydroascorbate reductase (MDHAR; EC1.6.5.4) activity was estimated as described by Miyake and Asada [41]. The change in absorbance of both the reaction mixtures was determined spectrophotometrically at 340 nm for 3 min, with the activity expressed as EU mg^−1^ protein.

Dehydroascorbate reductase (DHAR; EC 1.8.5.1) was determined by the protocol outlined by Nakano and Asada [39] and the OD noted at 265 nm for 3 min. The activity of DHAR was presented as EU mg^−1^ protein.

The levels of glutathione (GSH) and ascorbate (AsA) were estimated according to the methods of Yu [42] and Huang et al. [43]. By subtracting oxidized glutathione from the total reduced glutathione, the content of glutathione was determined in the soybean plants.

### 2.7. Measurement of Methylglyoxal Content and Activity of GlyI and GlyII

The methylglyoxal (MG) levels were measured from the leaves using a method adopted by Wild et al. [44]. The OD was noted at 288 nm using a spectrophotometer (Beckman 640D, Brea, CA, USA) to estimate the levels of MG.

The activities of glyoxalase I (EC: 4.4.1.5) and glyoxalase II (EC: 3.1.2.6) were determined by the protocols provided by Hasanuzzaman et al. [45] and Principato et al. [46], respectively. The activity was revealed as μmol min^−1^ mg^−1^ protein.

### 2.8. Estimation of Total Phenols and Total Flavonoids

The estimation of phenolic content was carried out by the Folin–Ciocalteu reagent following the procedure outlined by Chun et al. [47] and phenolic content was presented as mg gallic acid equivalent (GAE) g^−1^ of extract. For the determination of flavonoid content, the procedure described by Zhishen et al. [48] was employed. Flavonoid content was presented as mg catechin equivalents g^−1^ of extract.

### 2.9. Estimation of Mineral Elements

The leaf samples (each 500 mg) were digested at 60 °C in a mixture of H_2_SO_4_ and HNO_3_ (1:5, v/v) and then cooled. Then the samples were used for the estimation of Na, K, and Ca using a flame photometer.

### 2.10. Statistical Analysis

Data for each attribute were analyzed using SPSS statistical software version 17.0 (SPSS Inc., Chicago, IL, USA) to work out analysis of variance. The mean values were compared for significant difference using the Duncan’s multiple range test at 5% probability. Each treatment was replicated 5 times.

## 3. Results

### 3.1. Growth and Biomass Yield

**Figure 1:**
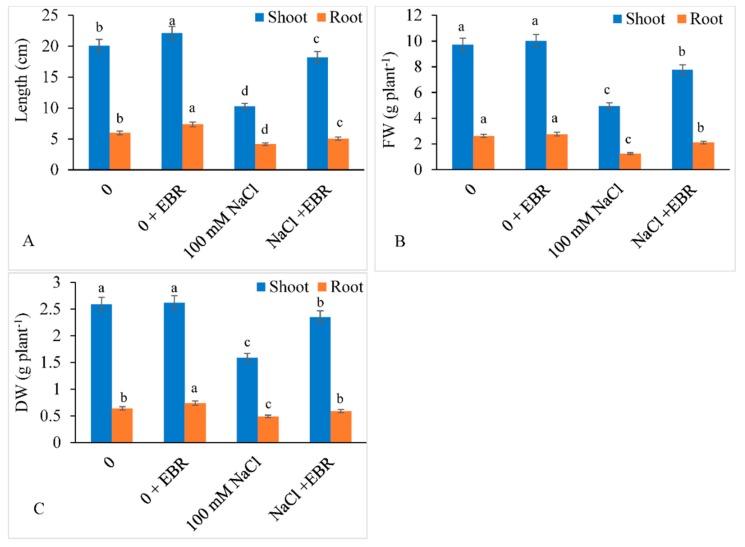
Ameliorating role of 24-epibrassinolide (EBR) on (**A**) length, (**B**) fresh weight, and (**C**) dry weight of shoot and root under NaCl toxicity in soybean. Data presented are the means ± SE (*n* = 5) and significant difference calculated between the means at *p* ≤ 0.05 using the Duncan’s multiple range test.

Shoot length declined by 48.95% and root length by 43.53% with NaCl stress. The shoot FW also decreased by 49.12% and root FW by 52.47% under saline stress relative to control. Moreover, the shoot and root DW decreased by 38.61% and 23.43%, respectively, with NaCl stress. Application of EBR induced the shoot height, root length, and FW of shoot and root of salt-stressed plants by 77.65%, 21.92%, 56.96%, and 68.80%, respectively, relative to the salt-stressed plants not treated with EBR. EBR enhanced the shoot DW by 47.75% and root DW by 10.93% relative to the salt-stressed plants that received no EBR treatment (Figure 1A–C).

### 3.2. Photosynthetic Pigments, Chlorophyll Fluorescence, and Leaf Gas Exchange Parameters

**Figure 2:**
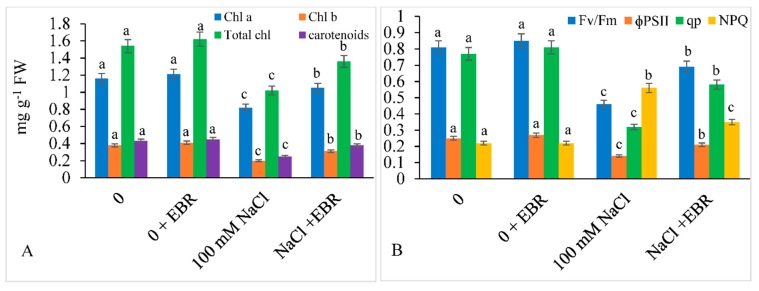
Effect of 24-epibrassinolide (EBR) on (**A**) pigment content and (**B**) chlorophyll fluorescence parameters under NaCl toxicity in soybean. Data presented are the means ± SE (*n* = 5) and significant difference between the means calculated at *p* ≤ 0.05 using the Duncan’s multiple range test.

In comparison to control, a decline of 33.76% in total Chl was observed in NaCl-treated plants (Figure 2A). However, EBR supplementation to the NaCl-treated plants showed improved pigment contents by 21.90%, 35.48%, and 25.00% in case of Chl a, Chl b, and total Chl, respectively. A similar declining trend was also observed for carotenoid content (41.86%) in salt-stressed plants with respect to control. Supplementation of NaCl-treated plants with EBR led to increased carotenoid content to an extent of about 52.00%. (Figure 2A).

All the parameters related to chlorophyll fluorescence showed a significant decline under NaCl treatment. Under salt stress conditions, reduction in PSII efficiency (*F*v/*F*m) by 43.20%, quantum yield of PSII (ΦPSII) by 44.00%, and photochemical quenching by 58.44% was observed compared to those in the control plants. Moreover, under the same conditions of salt stress, an increase of about 60.71% was observed in non-photochemical quenching (NPQ). Supplementing the salt treated plants with EBR led to increased *F*v/*F*m by 50.00%, ΦPSII by 50.00%, and qP by 81.25% compared to those in the plants not treated with EBR. However, a decrease of 37.50% was observed in case of NPQ (Figure 2B).

**Figure 3:**
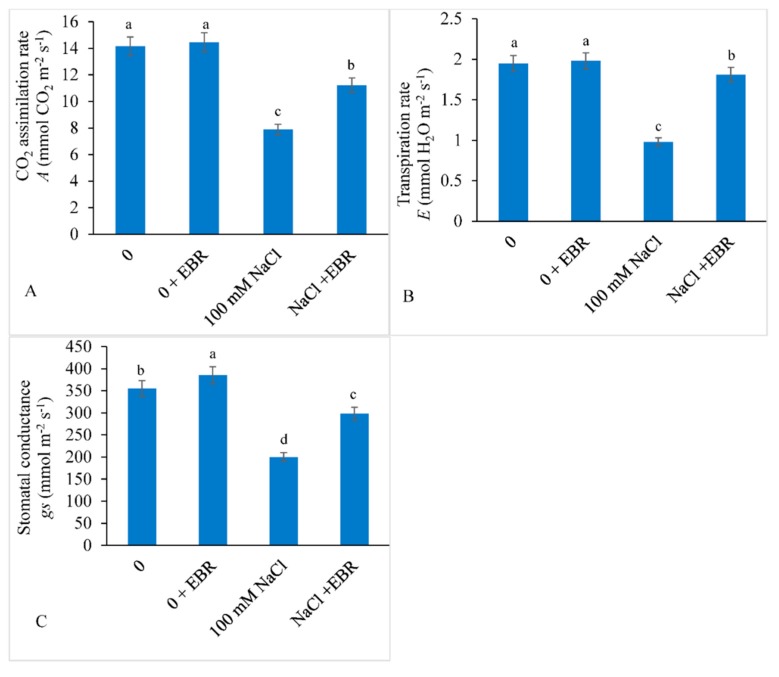
Effect of exogenously applied 24-epibrassinolide (EBR) on (**A**) CO_2_ assimilation rate (*A*), (**B**) transpiration rate (*E*), and (**C**) stomatal conductance (*g_s_*) in soybean under NaCl toxicity. Data presented are the means ± SE (*n* = 5) and significant difference between the means calculated at *p* ≤ 0.05 using Duncan’s multiple range test.

The gas exchange parameters such as CO_2_ assimilation rate (*A*), transpiration rate (*E*), and stomatal conductance (*g_s_*) decreased by 44.24%, 49.74%, and 43.66%, respectively, with NaCl stress over control. However, NaCl-treated plants supplemented with EBR showed a lower decrease of 20.70% in *A*, 7.17% in *E*, and 16.05% in *g_s_* relative to those in control (Figure 3A–C).

### 3.3. Proline and GB

**Figure 4:**
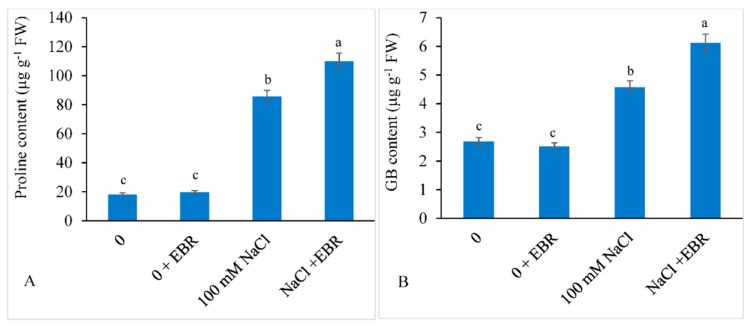
Exogenously applied 24-epibrassinolide (EBR) enhanced the proline (**A**) and glycine betaine (GB) (**B**) content in soybean under NaCl stress. Data presented are the means ± SE (*n* = 5) and significant difference between the means calculated at *p* ≤ 0.05 using the Duncan’s multiple range test.

The NaCl stressed soybean plants showed enhanced proline by 4.68-fold and GB by 1.70-fold compared to those in the control plants. External application of EBR to NaCl stressed plants further elevated the accumulation of proline and GB content by 1.28-fold and 1.33-fold, respectively, relative to those in salt-stressed plants not treated with EBR (Figure 4A,B).

### 3.4. H_2_O_2_ and MDA Content and EL

**Figure 5:**
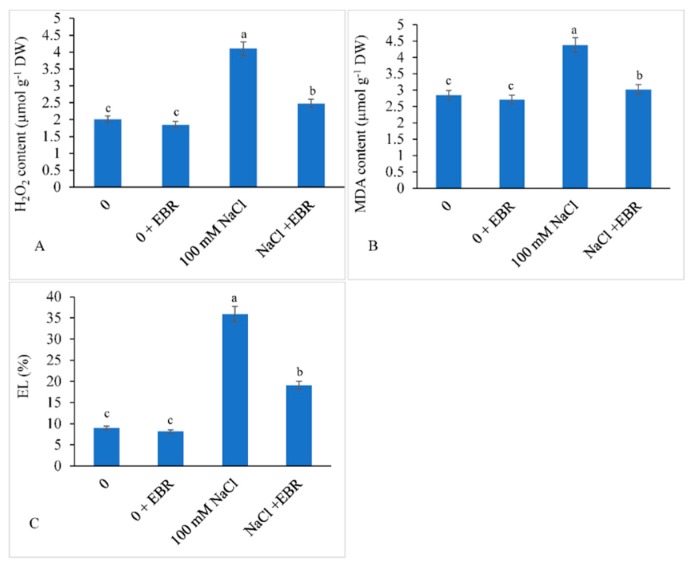
Exogenously applied 24-epibrassinolide (EBR) reduced the H_2_O_2_ (**A**) and malondialdehyde (MDA) (**B**) contents as well as electrolyte leakage (EL) (**C**) in soybean plants under NaCl stress. Data presented are the means ± SE (*n* = 5) and significant difference between the means calculated at *p* ≤ 0.05 using the Duncan’s multiple range test.

The soybean plants treated with NaCl showed enhanced H_2_O_2_ by 1.95-fold, MDA by 1.53-fold, and EL by 3.99-fold relative to those in the control plants. Salt-stressed plants supplied with EBR showed less accumulation by 1.08-fold, 1.05-fold, and 2.12-fold in H_2_O_2_, MDA, and EL over the controls (Figure 5A–C).

### 3.5. Activities of Antioxidant Enzymes and Enzymes of Ascorbate-Glutathione Cycle

**Figure 6:**
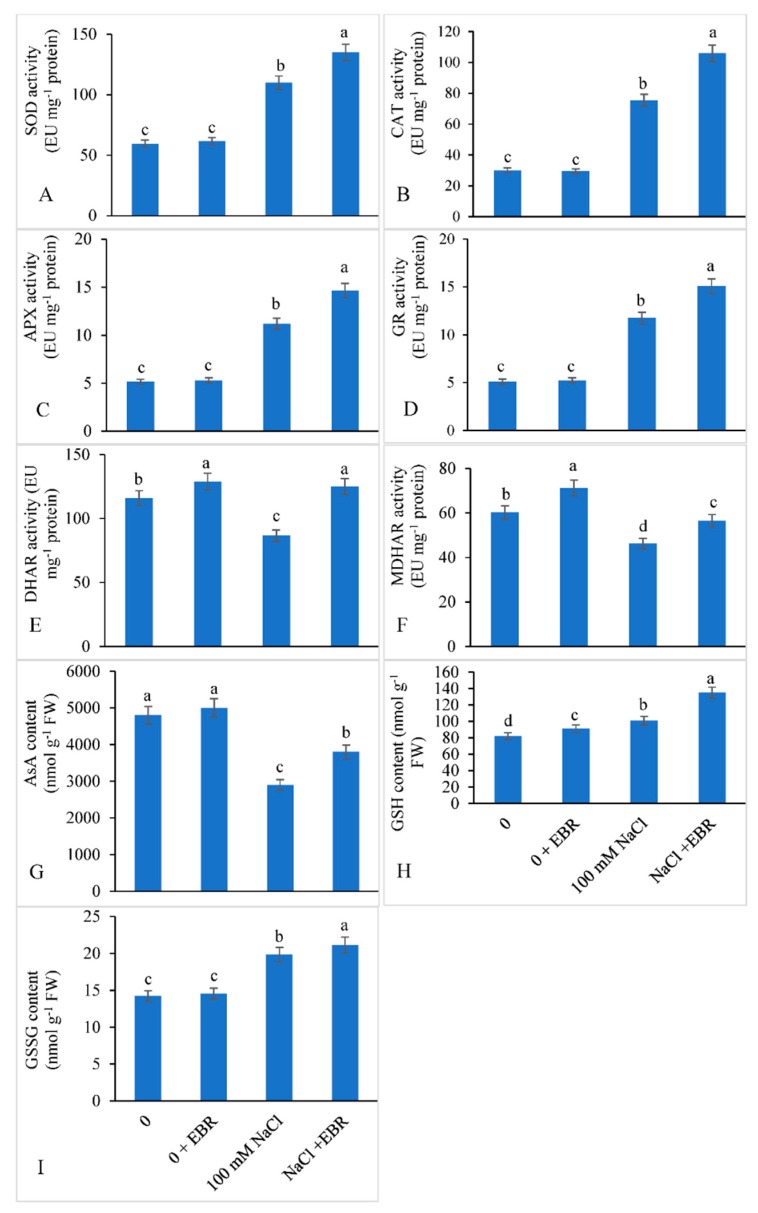
Exogenously applied 24-epibrassinolide (EBR) enhanced the activities of (**A**) superoxide dismutase (SOD), (**B**) catalase (CAT), (**C**) ascorbate peroxidase (APX), (**D**) glutathione reductase (GR), (**E**) Dehydroascorbate reductase (DHAR), (**F**) manodehydroascorbate reductase (MDHAR), (**G**) ascorbate (AsA) content, (**H**) glutathione (GSH) content, and (**I**) GSSG content in soybean plants under NaCl stress. Data presented are the means ± SE (*n* = 5) and significant difference between the means calculated at *p* ≤ 0.05 using the Duncan’s multiple range test.

The impact of NaCl stress on the activities of all antioxidant enzymes are shown in Figure 6A–F. The NaCl-treated plants showed enhanced activities of SOD by 84.87%, CAT by 150.94%, APX by 117.24%, GR by 129.49%, DHAR by 25.36%, and MDHAR by 23.25% over the respective controls. EBR application to NaCl stressed plants further enhanced the activities of SOD, CAT, APX, GR, DHAR, and MDHAR by 22.72%, 40.28%, 30.77%, 28.34%, 44.42%, and 22.20%, respectively, compared with those in the salt-stressed plants receiving no EBR.

Saline stress decreased AsA content by 39.58% and increased GSH by 23.17% and GSSG by 39.38% compared with the controls (Figure 6G–I). Salt-treated plants supplied with EBR increased the AsA, GSH, and GSSG by 31.03%, 33.66%, and 6.71% relative to those in salt-stressed plants supplied with no EBR.

### 3.6. Glyoxalase System

**Figure 7:**
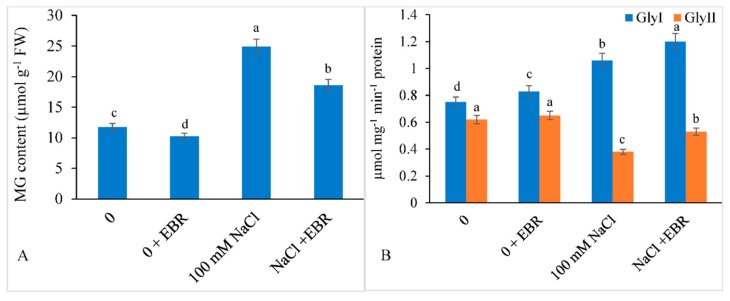
24-epibrassinolide (EBR) declines methylglyoxal (MG) content (**A**) and enhances GlyI and GlyII (**B**) under NaCl toxicity in soybean. Data presented are the means ± SE (*n* = 5) and significant difference between the means calculated at *p* ≤ 0.05 using the Duncan’s multiple range test.

In comparison with the control plants, salt treatment led to increased accumulation of MG by 111.67%, however, the treatment NaCl + EBR showed 58.55% less accumulation of MG (Figure 7A).

The activity of GlyI increased by 41.33% and that of GlyII decreased by 38.70% under salt stress. EBR supplemented NaCl-treated plants showed enhanced activity of GlyI (60.00%) and less decrease in GlyII (14.51%) compared to the controls (Figure 7B).

### 3.7. Total Phenols and Total Flavonoid Content

**Figure 8:**
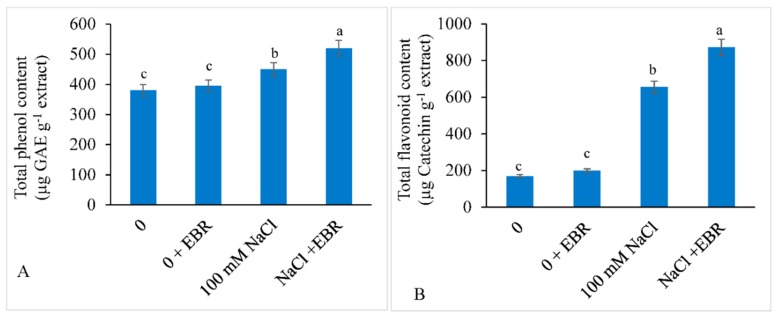
Effect of 24-epibrassinolide (EBR) on (**A**) total phenol content and (**B**) total flavonoid content in soybean plants under NaCl stress. Data presented are the means ± SE (*n* = 5) and significant difference between the means calculated at *p* ≤ 0.05 using the Duncan’s multiple range test.

Saline stress increased the total phenol content by 18.42% and total flavonoid content by 285.29% relative to the control. Exogenously supplied EBR further enhanced the total phenol and total flavonoid contents by 15.55% and 33.12%, respectively, over controls, i.e., salt treated plants which were not fed with EBR (Figure 8A,B).

### 3.8. Mineral Elements

**Figure 9:**
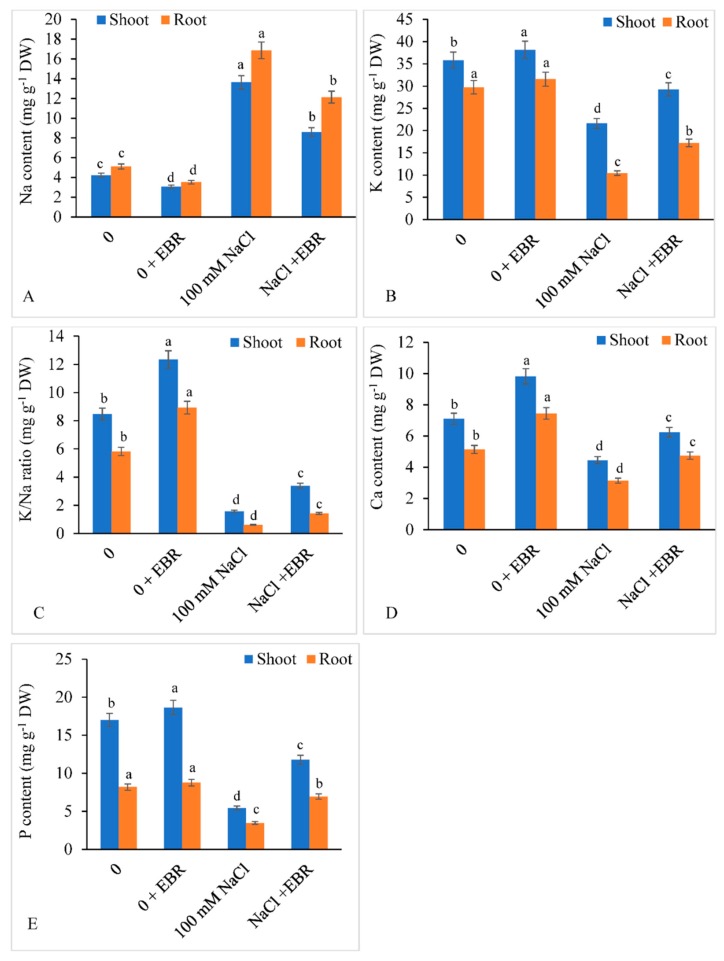
Effect of 24-epibrassinolide (EBR) on shoot and root mineral contents (**A**) Na content, (**B**) K content, (**C**) K/Na ratio, (**D**) Ca content, and (**E**) P content in soybean plants under NaCl stress. Data presented are the means ± SE (*n* = 5) and significant difference between the means calculated at *p* ≤ 0.05 using the Duncan’s multiple range test.

Saline stress resulted in a significant decline in the concentrations of key mineral elements in both shoot and root. Salt stress resulted in enhanced accumulation of Na in shoot by 481.13% and in root by 508.74% over the controls. The shoot K, Ca, P, and K/Na ratio decreased by 44.17%, 39.63%, 68.11%, and 90.40%, respectively, over salt-stressed plants fed with no EBR salt stress also reduced root K, Ca, P, and K/Na ratio by 50.48%, 78.31%, 57.63%, and 91.92% over the respective controls. However, NaCl stressed plants supplemented with EBR elevated the shoot and root mineral elements and restricted the accumulation of Na ions over controls (Figure 9A–E).

## 4. Discussion

The current study showed a significant decline in growth and biomass yield of soybean plants under saline stress, and these findings corroborate with those of Rasool et al. [19] in chickpea. Many other authors also reported harmful effect of NaCl on growth and biomass yield [49,50,51]. The reason might have been that NaCl stress restricted the division and elongation of cells and also decreased mineral uptake [49,52,53]. EBR has been investigated to exalt growth in *Capsicum annuum* (Abbas et al. [54]), *Pisum sativum* (Shahid et al. [55]), and *Solanum lycopersicon* (Ahmad et al. [7]). Hayat et al. [56] have reported enhancement in photosynthetic rate and carbonic anhydrase activity in salt-stressed *Vigna radiata* supplemented with 28-homobrassinolide thereby showing enhanced growth. In fact, EBR is believed to activate H^+^-ATPase which is involved for cell wall loosening enzyme activation, one of the major factors for growth promotion [57]. EBR-induced growth promotion can be attributed to the role of EBR in modulating cellulose biosynthesis, and increasing the rates of cell division and cell elongation [58].

Overall performance of plants is reflected by their photosynthetic potential that can be exhibited through several growth and physiological related parameters. Salt stress has been reported to adversely influence chlorophyll content, leading to increased degradation of pigments and impaired biosynthesis [59]. Salt stress in plants is often accompanied with the destabilization of chlorophyll protein complex associated pigments more often manifested in reduced photosynthetic pigments due to augmented chlorophyllase activities [19]. Supplementation of plants with EBR under salt stress conditions enhances their photosynthetic potential. EBR-induced increase in chlorophyll synthesis in plants under salt stress may be attributed to plants’ regulatory role against an abiotic stress [60]. Under salt stress conditions, elevation in photosynthesis due to enhanced enzyme activities of the Calvin cycle modulated through increased expression of BR biosynthetic pathway has also been reported [61]. Several researchers have also reported an increase in enzyme activities related to carbon metabolism in salt-stressed plants supplemented with EBR, because EBR is believed to reduce the Na^+^ ion uptake under salt stress conditions. Our results corroborate with those for different crops, e.g., *Oryza sativa*, *Brassica juncea, Cicer arietinum*, and *Vigna radiata*, [49,56,62,63]. Moreover, supplementing salt-stressed plants with EBR can assist mineral uptake, especially Mg^2+^, which possibly explains the elevated levels of pigments under stressful environments. In *Raphanus sativus*, an increase in photosynthetic pigments was also observed under salt stress when supplemented with EBR [64]. In photosynthetic reaction center, carotenoids play a prominent role through regulation of photo-protection against auto-oxidation [65]. During salt stress, elevated carotenoid biosynthesis may be attributed to the antioxidant activity of carotenoids that assist plants to overcome oxidative stress [53]. Enhanced biosynthesis of carotenoids under salt stress conditions with EBR supplementation is likely due to a key enzyme, phytoene synthase (PSY) in carotenoid biosynthetic pathway [28].

An alternative method extensively implied to assess stress tolerance and acclimation of plants to several environmental cues is chlorophyll fluorescence [66]. In the present study, a decrease in *F*v/*F*m was observed under salt stress; these findings are in agreement with those reported for Indian mustard [67]. Under salt stress conditions, a significant decrease was also observed in ΦPSII and qP and our reports are analogous to those for *Solanum melongena* [68] and *Vigna unguiculata* [69]. Salt stress has been reported to lead to impaired PSII electron flow [70] which in turn can cause photoinhibition that upshot the obliteration in antenna molecules. At the PSII acceptor side, electron transport from primary to secondary acceptor is blocked that drastically reduces *F*v/*F*m under saline regimes [71]. However, during the present study, the salt-induced negative effects on various photosynthetic parameters like ΦPSII, qP, *F*v/*F*m ratio, and NPQ were reversed when the salt-stressed soybean plants were supplemented with EBR. These findings can be supported by the findings of Fariduddin et al. [72,73] who have reported a similar effect in salt treated cucumber and Indian mustard, respectively. Under salt stress, brassinosteroids are known to help plants effectively to uphold their electron pool which results in reduction of PSII photoinhibition and to maintain ΦPSII, *F*v/*F*m ratio, and qP. Similar findings have been reported in eggplant [68] and wheat [74,75]. During the present study, a decline in NPQ was observed in the salt-stressed soybean plants supplemented with EBR. These findings advocate that EBR helps maintain the integrity of thylakoid membranes and at the same time prevents PSII from over-excitation.

Decreased CO_2_ assimilation rate (*A*), transpiration rate (*E*), and stomatal conductance (*gs*) with saline stress are analogous to the findings of Parveen and Ashraf [76] in *Zea mays*. Salt-stressed plants exhibited reduced leaf gas exchange parameters similar to what has been documented by Fariduddin et al. [72] and Wang et al. [77] in *Cucumis sativus*, and Hayat et al. [56] and Mahmood et al. [78] in *Vigna radiata*. Salt stress is believed to induce alteration in enzyme activities which leads to protein dysfunction, which might be the main reason for decreased leaf gas exchange parameters. However, supplementation of EBR enhanced the leaf gas exchange attributes as has been already investigated by Hu et al. [79] in *Capsicum annuum* and Lima and Lobato [69] in cowpea. It has been reported that EBR significantly enhanced the CO_2_ assimilation rate [80] and Rubisco enzyme activity [42], thereby increasing photosynthetic activity, which in turn increased plant growth and development.

Proline and GB have been reported as potential osmolytes whose accumulation is enhanced due to salt stress [81,82] as has been earlier reported in different plants, e.g., in *Linum usitatissimum* (Khan et al. [83]), *Morus alba* (Ahmad et al. [84]), *Vigna radiata* (Khan et al. [13]), and *Oryza sativa* (Hasanuzzaman et al. [85]). The main role of proline and GB is believed to be the cell osmoregulation under saline stress, thereby protecting the cell from osmotic stress [13,53]. Proline has multiple functions like acting as a molecular chaperone, storing energy, preventing membrane damage, and enhancing enzyme activity [86,87]. Proline also acts as an efficient antioxidant because it effectively helps quench ROS [88]. Chen and Murata [89] reported that GB helps in osmotic adjustment and protects cellular constituents and protein damage during an abiotic stress. Ohnishi and Murata [90] reported that GB plays a great role in protecting photosynthetic apparatus and enzyme activity. In our study, supplementation of EBR further enhanced the proline content similar to what has been reported by Choudhary et al. [91] and Ramakrishna and Rao [92] in *Raphanus sativus*. In wheat, salt-stressed plants supplemented with EBR showed enhancement in proline content and also in proline metabolizing enzymes [93]. The enhanced proline accumulation might have been due to the enhanced activity of enzymes related to synthesis of proline and/or reduction in catabolizing enzymes [18,94]. Proline and GB syntheses restore the photosynthetic efficiency, improve growth, and decrease oxidative damage [83,85]. For example, externally applied EBR enhanced the proline content in different plants under different types of abiotic stresses, e.g., in mung bean during aluminium stress [95], in mustard under copper stress [96], and in peach tree under cold stress [97]. Elevated proline content with EBR may be attributed to change in P5CS enzyme (D1-pyrroline-5-carboxylate) responsible for activation of the proline synthesis pathway and decline in the activity of proline dehydrogenase [97].

Saline stress increased the H_2_O_2_ and MDA contents and EL in the current investigation; this is similar to what has been found in wheat (Zheng et al. [98]). In another study, chickpea plants subjected to NaCl stress also showed increased levels of H_2_O_2_ and MDA contents as well as EL [19,53,99]. EBR treatment decreased the H_2_O_2_ and MDA contents as well as EL in the current study. These findings are similar to what has been recorded in rice [22,62] and tomato [100]. EBR also provided protection to peanut membranes subjected to iron deficiency [101], and also decreased the H_2_O_2_ production and accumulation of MDA and EL in Hg-stressed chickpea seedlings [58]. The decline in lipid peroxidation due to EBR might have been due to increased levels of endogenous growth hormones and their interhormone cross-talk [73].

Activities of antioxidant enzymes, such as SOD and CAT, increased under NaCl stress in the present study. Similar findings have been reported by other scientists in different plants [53,56,102]. SOD is believed to accelerate the conversion of superoxide anion (O_2_^−^) into molecular oxygen (O_2_) and H_2_O_2_. The H_2_O_2_ produced is still toxic and is acted by catalase to convert it into oxygen and H_2_O [103]. EBR further enhanced the activities of SOD and CAT to provide more tolerance to the soybean plants against NaCl stress and similar results have been recorded by Ahmad et al. [7] in tomato. Shahbaz and Ashraf [104] found that externally supplied EBR to NaCl-stressed wheat plants caused enhanced activities of catalase and peroxidase. The mitigating role of BRs against salt stress has also been described by Anuradha and Rao [105] in *Oryza sativa*, Ali et al. [63] in *Cicer arietinum*, El-Mashad and Mohamed [106] in *Vigna sinensis,* and Çoban and Göktürk Baydar [107] in *Mentha piperita.* Song et al. [108] observed that supply of BRs to salt treated *Cucumis sativus* up-regulated the activities of SOD and CAT coupled with the reduction in MDA content and electrolyte leakage.

In plants, the AsA-GSH pathway comprised both enzymatic and non-enzymatic antioxidants, which play a crucial role in quenching the ROS and protecting the cell from the damaging effects of highly oxidizing ROS. The AsA-GSH pathway operates in cytosol, mitochondria, plastids, and peroxisomes, and it comprises four key enzymes, i.e., ascorbate peroxidase (APX), manodehydroascorbate reductase (MDHAR), dehydroascorbate reductase (DHAR), and glutathione reductase (GR), and two antioxidant metabolites, ascorbate (AsA) and glutathione (GSH) [109,110]. In this cycle, APX reduces H_2_O_2_ to water, yielding MDHA as an unstable initial product. MDHA in turn gets dismutated to ascorbate and DHA, which then are reduced by GSH to GSSG by DHAR. GR uses NADPH produced principally from photosynthesis to regenerate GSH from oxidized GSSG [8,109]. In the present study, the enhanced GR activity in salt-stressed soybean plants supplemented with EBR helps them to maintain NADP^+^ pool for electron transport and also helps them to protect photosynthetic machinery from photooxidation [56]. For normal cell functioning, EBR supplementation is useful in maintaining the GSH/GSSG ratio [111]. Antioxidants like glutathione and ascorbate thwart plasma membrane oxidation and also serve as redox buffering agents [109]. Ascorbate and glutathione works together in AsA-GSH cycle to metabolize ROS [112]. During the present study, activities of MDHAR and DHAR, as well as GSH/GSSG ratio showed a significant decline under salt stress; nevertheless, EBR re-established and enhanced the activities of the enzymes. EBR improved AsA and GSH synthesis by activating MDHAR and DHAR enzymes, thereby providing a determined supply of GSSG to GR and AsA to APX [100]. EBR supplementation augmented DHAR and MDHAR activities in the soybean plants. Such findings have also been reported in *Acacia gerrardii* [113], *Solanum lycopersicum* [114], and *B*. *juncea* [115]. In comparison to control plants, salt-treated soybean plants supplemented with EBR demonstrated enhanced DHAR and MDHAR activities, advocating that through the H_2_O_2_ reduction to water by APX, the radicals generated were transported instantly back to AsA via MDHAR or by an unprompted disproportion processes [114]. GSH plays a substantial part in maintaining GSH/GSSG ratio in the transformation of GSSG to GSH. Application of EBR improved production of GSH, which transformed more GSSG to its reduced form and generate a reduced redox homeostatic environment. Taken together, our study concluded that under salt stress EBR has the potential to modulate the AsA-GSH cycle to a redox state that plays a fundamental role in stress tolerance of soybean plants.

Accumulation of MG in plants is deliberated as a key strategy to overcome salt stress conditions [116,117,118]. During the present study, we observed higher levels MG in salt-stressed soybean plants. Enhanced levels of MG may have detrimental effects on plants or led to the deprivation of GSH pool owing to its conversion to hydroxyacylglutathione [119]. However, higher levels of GlyI and GlyII were observed in the salt-stressed soybean plants supplemented with EBR, thereby perhaps leading to the protection of these plants against salt stress-induced accumulation of MG [116]. In response to a stress, higher MG levels have also been reported in *Oryza sativa* and *Vigna radiata* [50,120]. Over-expression of GlyI and GlyII in transgenic plants displayed higher levels of MG subsequently decreasing lipid peroxidation via GSH detoxification [121]. Several researchers have reported enhanced activities of GlyI under salt stress conditions in various plant species [116,119]. Due to proteolytic degradation of enzymes, a decline in GlyII activity under salt toxicity has also been documented. It has also been observed that under high temperature stressed *Ficus concinna* plants supplemented with EBR maintained the pool of GlyI and GlyII enzymes [122]. EBR might enhance mineral uptake and modulate endogenous hormone levels that are chiefly concerned with upkeeping of the glyoxalase pool and MG detoxification against stressful conditions. EBR alleviates salt-induced oxidative stress through the maintenance of GlyI and GlyII activities, demonstrating that EBR might facilitate GSH refurbishment and glutathione redox potential via the glyoxalase system.

Plants tolerate a stress by accumulating high flavonoid contents [123]. They act as potential antioxidants to scavenge the ROS (Michalak [124]). Ben Abdallah et al. [125] and Taïbi et al. [126] also reported increased flavonoid content in *Solanum nigrum* and *Phaseolus vulgaris,* respectively, under NaCl stress. Salt-induced oxidative stress modulates flavonoid pathway to synthesize more content of flavonoids [127]. Nijveldt et al. [128] reported that flavonoids prevent the activity of lipoxygenase enzyme, thereby hampering the conversion of polyunsaturated fatty acids to oxygen-containing derivatives. Potapovich and Kostyuk [129] reported that flavonoid accumulation under stress provides protection to membranes as it helps decrease lipid peroxidation. EBR supplementation further enhanced the flavonoid contents in *Tinospora cordifolia* [130] and *Camellia sinensis* [131]. Phenolics are non-enzymatic antioxidants and they help in quenching free radicals [124,132,133]. Similar results of increase in phenolics under salt stress have also been reported by Mehr et al. [134] in *Anethum graveolens*, Tomar and Agarwal [133] in wheat, Dawood and EL-Awadi [135] in faba bean. The enhanced levels of phenolics under stress conditions might be due to increased enzyme activity connected with their synthesis [136]. Supplementation of EBR further enhanced the phenolic content to provide tolerance against salinity stress in the present study.

During NaCl stress, the cumulation of Na^+^ increased. It is similar to what has been reported by De Leon et al. [137] in *Oryza sativa* and Gu et al. [138] in *Brassica oleracea*. High accumulation of Na^+^ inhibits absorption of basic elements like Ca^2+^, K^+^, etc., thereby causing ion imbalance. Such restricted uptake of essential elements could be the main reason for the decreased growth and biomass yield and induction of osmotic and oxidative stress. Supplementation of EBR maintained the mineral ions and regulated the uptake of Na^+^ and promoted the uptake of essential elements.

## 5. Conclusions

Soybean plants exposed to NaCl stress showed a considerable decrease in growth, biomass yield, chlorophyll content, chlorophyll fluorescence parameters, and leaf gas exchange parameters. However, external supplementation of EBR enhanced all these parameters to an appreciable level. Proline and GB increased under NaCl stress and further enhancement was observed with EBR supplied externally. The oxidative stress biomarkers and methylglyoxal level also increased with NaCl stress, but they decreased in plants supplemented with EBR. The activities of antioxidant enzymes and the enzymes involved in the Asc-Glu cycle and glyoxalase system were found to be enhanced by the application of EBR. The external supplementation of EBR enhanced the essential mineral uptake accompanied with a decreased Na accumulation in shoot and root. Overall, the application of EBR mitigated the NaCl toxicity in soybean plants and thus it can be applied to other plants as well. Such a sustainable approach can be used to achieve enhanced crop production under salt-affected soils. However, further investigation like molecular approach is needed to unveil the real mechanism of EBR action.
